# Real-world efficacy and safety of burosumab in tumor-induced osteomalacia: case series from an early access program

**DOI:** 10.1093/jbmrpl/ziaf039

**Published:** 2025-03-10

**Authors:** Simon Cadiou, Roland Chapurlat, Guillaume Couture, Pascal Guggenbuhl, Pascale Guillot, Rose-Marie Javier, Nadia Mehsen, Caroline Morizot, Sophie Trijau, Julien Paccou

**Affiliations:** Department of Rheumatology, University Hospital of Rennes, 35700 Rennes, France; INSERM UMR_S 1033, University Claude Bernard-Lyon, E. Herriot Hospital, 69000 Lyon, France; Department of Rheumatology, University Hospital of Toulouse, 31000 Toulouse, France; CHU Rennes, University Rennes, INSERM, Institut NuMeCan (Nutrition Metabolism and Cancer), 35700 Rennes, France; Department of Rheumatology Hôtel-Dieu, CHU 44000 Nantes, France; Department of Rheumatology, Strasbourg University Hospital, 67000 Strasbourg, France; Department of Rheumatology, Groupe Hospitalier Pellegrin—CHU, 33000 Bordeaux, France; Department of Rheumatology, Nancy University Hospital, 54000 Nancy, France; AP-HM Hôpital ste Marguerite, 13000 Marseille, France; Department of Rheumatology, MABlab ULR 4490, CHU Lille, University of Lille, 59000 Lille, France

**Keywords:** Tumor-induced osteomalacia, Fibroblast Growth Factor 23, hypophosphatemia, burosumab, phosphaturic tumor

## Abstract

Tumor-induced osteomalacia (TIO) is a rare paraneoplastic syndrome due to a phosphaturic tumor, which overproduces FGF23, causing hyperphosphaturia, hypophosphatemia, low 1,25(OH)2D, and osteomalacia. Complete surgical resection is the standard of care, but some tumors cannot be found, and others cannot be removed. In such difficult situations, burosumab, a fully human monoclonal antibody that targets and inhibits excess circulating FGF23, is a treatment option. Early access program (EAP) to burosumab has been established for patients with TIO in France in July 2022. Before that, access to burosumab at no cost on compassionate grounds was provided for a few patients. Between July 21, 2022 and December 3, 2023, an EAP was initiated for burosumab across 10 University Hospital Centers. The program included 9 patients (3 pre-exposed and 6 burosumab-naïve patients). The EAP included assessments of phosphatemia, pain levels using the visual analogue scale, and quality of life using the Routine Assessment of Patient Index Data 3 questionnaire. Patients’ ages ranged from 33 to 62 yr, with various BMI categories. Seven patients had at least 1 follow-up visit (3 pre-exposed and 4 burosumab-naïve patients). In the burosumab-naïve group, phosphatemia levels improved in 2 patients, with 1 achieving levels >0.8 mmol/L. Pain reduction was reported in all 4 naïve patients with follow-up, while pain levels in pre-exposed patients remained stable or fluctuated. Quality of life scores indicated minimal impairment or remission in 6 patients at baseline. No serious adverse events were observed. These preliminary findings following burosumab EAP for patients with TIO in France support benefits in terms of efficacy, safety, and ease of treatment. Burosumab appears to be a promising option for patients who are ineligible or refractory to surgery.

## Introduction

Tumor-induced osteomalacia (TIO), also defined as oncogenic osteomalacia, is a rare acquired paraneoplastic syndrome characterized by defective mineralization of bone and cartilage in children, and bone in adults.^1^ This condition results from the overproduction of FGF23 by tumors. FGF23 is a key regulator of phosphate homeostasis that decreases renal phosphate reabsorption in the proximal tubules and inhibits kidney 1α-hydroxylase activity, leading to reduced active vitamin D levels and subsequent decreased intestinal phosphate absorption.^2^^,^^3^

The primary biochemical markers of TIO include chronic hypophosphatemia and abnormally low levels of 1,25(OH)2D. Clinically, patients may present with fractures, pseudo-fractures, fatigue, musculoskeletal pain—often localized in weight-bearing areas—and severe myopathy. These symptoms significantly impair health-related quality of life (HR-QoL) due to rapid clinical deterioration. Diagnosing TIO is challenging, often leading to misdiagnosis or delayed diagnosis, as the tumor can be difficult to detect and, in some cases, may not be localizable, which further complicates the identification and treatment process.^4^

When tumors are identifiable and accessible, surgical resection is the preferred and most effective treatment, leading to normalization of FGF23 levels and biochemical parameters. For patients with comorbidities or inoperable tumors, alternative treatments such as radiotherapy, cryoablation, or radiofrequency ablation may be employed.^1^^,^^4–6^

For patients who cannot access treatment, conventional therapy with phosphate supplements and active vitamin D analogues remains a commonly used alternative.

Burosumab, a fully human monoclonal antibody that targets and inhibits excess circulating FGF23, which has initially been approved for XLH rickets, has recently gained approval for TIO in Japan and Korea in 2019, in the United States in 2020 (US),^7^^,^^8^ and France since 2024.

The approvals of burosumab were supported by 2 phase 2 clinical trials conducted in the US^9^ and Japan and Korea.^10^ An open-label phase 2 trial (UX023T-CL201; NCT02304367) involving 14 adults with TIO demonstrated that 48 wk of burosumab treatment corrected hypophosphatemia, improved histomorphometric measures in bone biopsies, enhanced healing of fractures and pseudo-fractures, and reduced the occurrence of new fractures. Additionally, patients reported improvements in pain, fatigue, and HR-QoL.^9^ A phase 2 multicenter, open-label study in Japan and Korea (KRN23-002; NCT02722798) involved intraindividual dose adjustments of burosumab (ranging from 0.3 to 2.0 mg/kg every 4 wk) in 13 patients with TIO. Following the initial administration of burosumab, the mean serum phosphate levels in these patients increased and remained within the normal range from wk 14 through wk 112.^10^

On July 21, 2022, the French *Haute Autorité de Santé* (HAS) granted early access authorization for injectable burosumab in 10, 20, and 30 mg solutions. This authorization is for treating FGF23-related hypophosphatemia in pediatric patients aged 1 yr and older, and adults with TIO associated with phosphaturic mesenchymal tumors that cannot be resected or localized. The establishment of an early access program (EAP) for TIO patients treated with burosumab is a unique initiative, providing early therapeutic access while simultaneously collecting real-world data to better understand the disease, monitor treatment outcomes, and optimize patient care.

Indeed, evaluating the efficacy and safety of burosumab in TIO remains challenging due to the limited number of patients.

Beyond open label studies, case reports documenting the use of burosumab in TIO have been published but real-world evidence on its efficacy and safety in clinical practice is missing.^11^^,^^12^ The goal of this unique case series, conducted through a French EAP, was to generate real-world evidence on the treatment’s efficacy and safety in routine clinical practice.

## Materials and methods

### Program design

Early access programs,^13^^,^^14^ provide a pathway for patients who have exhausted available therapeutic options to gain temporary access to medications not yet authorized for specific therapeutic indications.

**Figure 1:**
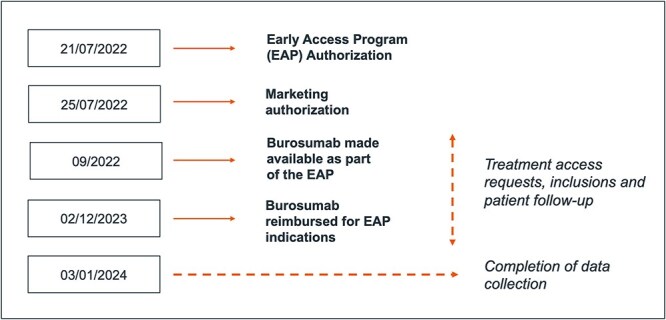
Early access program timeline.

On July 21, 2022, an EAP for burosumab was authorized in France for treating FGF23-related hypophosphatemia in patients with TIO aged 1 yr and older (Figure 1). Treatment access was restricted to physicians specializing in metabolic bone diseases. Physicians submitted an access form, approved by the hospital pharmacist, for validation. If approved, the treatment was initiated, and data was collected at baseline, every 2 mo for 6 mo, and then every 3 mo thereafter.

Patients were included prospectively between July 21, 2022 and December 3, 2023 for the assessment of clinical and biochemical characteristics before burosumab initiation as well as disease outcome. These were adults referred to tertiary Rheumatology Centers with TIO that cannot be resected or localized.

### Eligibility criteria

#### Inclusion criteria

To qualify for early access, patients must meet all of the following criteria:

- Age ≥1 yr, with a confirmed diagnosis of TIO, validated by a multidisciplinary expert panel.
- The tumor must be unresectable or partially resectable.
- Non-localizable tumor based on medical imaging, including 68Ga-DOTATOC PET/CT scans.
- Fasting serum phosphate concentration <0.80 mmol/L within the last 3 mo.
- Intact serum FGF23 (iFGF23) concentration ≥100 pg/mL over the past 3 mo.

#### Exclusion criteria

Patients are considered ineligible for early access if they meet any of the following conditions:

- Known hypersensitivity to the active substance or any of the excipients.
- Concurrent use of oral phosphate supplements or active vitamin D analogues.
- Elevated fasting serum phosphate levels (above the age-specific normal range), due to the risk of hyperphosphatemia.
- Presence of severe renal insufficiency or end-stage renal disease.

### EAP protocol

#### Physicians

By December 3, 2023, 10 physicians from 10 University Hospital Centers had submitted at least 1 request for burosumab treatment. Among these, 8 rheumatologists had at least 1 request approved. These 8 specialists were located in 8 different administrative regions.

#### Patients

As of December 3, 2023, all 9 approved patients had received at least 1 dose of burosumab (exposed patients). Among these, 3 patients had initiated burosumab treatment prior to the EAP request, with the treatment provided at no cost on compassionate grounds by the manufacturer, which had informed the French National Agency for the Safety of Medicines and Health Products. The remaining 6 patients were burosumab-naive.

Among the 9 exposed patients, seven had attended at least 1 follow-up visit (7 patients at mo 2, 4, and 6; 5 at mo 9; and 1 at mo 12).

#### Variables

In the context of the EAP for the use of burosumab, various variables were assessed to evaluate the treatment’s impact. A computerized registry was implemented to collect key variables in a standardized and mandatory manner, ensuring consistent and comprehensive data capture across all participants. Demographic information and detailed clinical data on the disease were collected for each patient. Laboratory data, including levels of serum phosphate, FGF23, PTH, and phosphaturia, were systematically recorded at baseline.

To evaluate the efficacy of burosumab, serum phosphate levels and patient-reported pain, measured using the Visual Analog Scale (VAS), were analyzed at treatment initiation, mo 2, mo 4, mo 6, and mo 9. Additionally, the assessment of Health-Related Quality of Life (HR-QoL) was conducted using the Routine Assessment of Patient Index Data 3 (RAPID-3) survey,^15^ as mandated by health authorities. The RAPID-3 questionnaire is a patient-reported outcome measure used in rheumatology to assess disease activity, focusing on physical function, pain, and overall health, without the need for laboratory tests. It provides a quick, easy, and patient-centered way to monitor disease severity and treatment effectiveness. This comprehensive approach allowed for a thorough evaluation of the clinical outcomes associated with burosumab treatment.

### Statistical analysis

Using SAS software (SAS Institute) version 9.4Statistical analyses followed ICH-E9 and French National Authority for Health (HAS) guidelines for EAP. Quantitative data were summarized using descriptive statistics: number of patients, mean, SD, median, first and third quartiles, and minimum and maximum values by treatment group and overall. Mean, median, and SD were reported with 1 additional decimal place, while quartiles, minimum, and maximum values retained their original precision. Qualitative data were presented as frequencies and percentages. No statistical tests were performed; only descriptive analyses were conducted.

## Results

### Patients’ characteristics

The baseline clinical and demographic characteristics of the 9 patients included as of December 3, 2023, are presented in Tables 1 and 2. These patients ranged in age from 33 to 62 yr and in weight from 54 to 116 kg. Four of the patients were female. The BMI distribution was as follows: 1 patient were underweight (BMI of less than 18 kg/m^2^), 2 patients had a normal BMI (between 18.5 and 25 kg/m^2^), 4 patients were overweight (BMI between 25 and 30 kg/m^2^), and 2 patients were obese (BMI between 35 and 40 kg/m^2^). Prior to the initiation of burosumab treatment, all patients had received conventional treatment (phosphate supplementation and active vitamin D) for durations ranging from 8 mo to 11 yr.

**Table 1:** Patients’ characteristics.

**Variables**	**Pre-exposed (*n* = 3)**	**Burosumab-naive (*n* = 6)**	**Total (*n* = 9)**
**Age at the access request visit (yr), mean (SD)**	57.3 (6.4)	46.7 (9.8)	50.2 (9.9)
**Sex, *n* (%)**			
**Female**	3 (100.0)	1 (16.7)	4 (44.4)
**Male**	0	5 (83.3)	5 (55.6)
**Height (cm), mean (SD)**	161.0 (13.7)	175.2 (8.0)	170.4 (11.7)
**Weight (kg), mean (SD)**	75.3 (21.5)	78.0 (20.5)	77.1 (19.5)
**BMI (kg/m** ^**2**^**), mean (SD)**	28.8 (6.3)	25.4 (6.2)	26.6 (6.1)
**Number of tumor locations, *n* (%)**			
**0**	0	3 (50.0)	3 (33.3)
**1**	1 (33.3)	3 (50.0)	4 (44.4)
**2**	2 (66.7)	0	2 (22.2)
**Tumor location(s), *n* (%)**			
**Thorax, lower limbs**	2 (66.7)	0	2 (22.2)
**Lower limbs**	0	2 (33.3)	2 (22.2)
**Head and neck**	0	1 (16.7)	1 (11.1)
**Spinal canal at D12-L1**	1 (33.3)	0	1 (11.1)
**Unidentifiable tumor**	0	3 (50.0)	3 (33.3)

Abbreviations: D12, 12th dorsal vertebra; L1, first lumbar vertebra.

**Table 2:** Laboratory data.

**Variables**	**Pre-exposed (*n* = 3)**	**Burosumab-naive (*n* = 6)**	**Total (*n* = 9)**
**Tests, mean (SD)**			
**Serum iPTH at initiation (pg/mL)**	*N = 3* 43.0 (28.1)	*N = 5* 86.5 (44.5)	*N = 8* 70.2 (43.2)
**Phosphaturia (mmol/24 h)**	*N = 1* 19.2	*N = 3* 52.1 (21.7)	*N = 4* 43.9 (24.2)
**Patient** ^**a**^	**FGF-23**	**FGF23 result on request for access**	
**1**	iFGF23^b^	148 pg/mL [8.2-54.3]	
**2**	cFGF23^c^	464 RU/mL [34.0-96.0]	
**3**	iFGF23^d^	357 pg/mL [22.7-93.1]	
**4**	iFGF23^b^	333 pg/mL [8.2-54.3]	
**5**	iFGF23^b^	161.6 pg/mL [8.2-54.3]	
**6**	iFGF23^d^	159.1 pg/mL [22.7-93.1]	

Abbreviations: iFGF23, intact fibroblast growth factor 23; cFGF23, C-terminal fibroblast growth factor 23.

a At inclusion, the value of the serum FGF23 was not available prior to burosumab initiation for the 3 pre-exposed patients (7-9).

b Manual immunoassays from Kainos.

c Manual immunoassays from Quidel-Immutopics.

d Automated chemiluminescence immunoanalyzers from Diasorin (Liaison XL).

At the time of diagnosis of TIO, 6 of the 9 patients had tumor localizations as follows: thorax and lower limbs (*n* = 2), lower limbs (*n* = 2), head and neck (*n* = 1), and spinal canal (*n* = 1). For the 3 remaining patients, tumors were unidentifiable.

Complete tumor resection was not achieved in some patients due to anatomical constraints or metastatic spread. In patient 2, resection of the femoral head lesion was limited by the risk of post-surgical necrosis. In patient 4, a second, inoperable spinal tumor was discovered post-surgery. Patient 5’s initial incomplete resection, due to a lack of diagnosis, could not be completed later without severe neurological risks. In patient 7, secondary pulmonary metastases were unresectable despite complete tibial tumor removal. Similarly, in patient 9, an incomplete patellar tumor resection led to pulmonary metastases, 1 of which was confirmed as FGF23-secreting. These cases highlight the surgical challenges associated with TIO.

The 6 patients who had not yet been treated with burosumab at the time of requesting access under the EAP exhibited recent serum phosphate levels below 0.8 mmol/L, with a median of 0.4 mmol/L (IQR: 0.4-0.5 mmol/L), and iFGF23 values of at least 100 pg/mL.

Specifically, laboratory data showed that among the burosumab-naive patients (*n* = 6), baseline serum phosphate levels ranged between 0.37 and 0.64 mmol/L, with FGF23 levels between 148 and 464 pg/mL (RU/mL for patient 2). Intact fasting PTH levels for these patients ranged from 25.1 to 138.2 pg/mL. Phosphaturia, measured in 3 of the naive patients, ranged from 27.4 to 68.0 mmol/24 h. For the pre-exposed patients (*n* = 3), serum phosphate levels were higher, ranging between 0.88 and 1.13 mmol/24 h. FGF23 assay data were not available at the time of the treatment request for these patients. Intact fasting PTH levels, available for 2 pre-exposed patients, were 22.0 and 75.9 pg/mL. Phosphaturia, measured in 1 pre-exposed patient, was 19.2 mmol/24 h.

Out of the 9 patients included, 8 reported experiencing pain. The distribution of pain severity was as follows: no pain (VAS 0), mild pain (2 patients; VAS 1-39), moderate pain (3 patients; VAS 40-59), severe pain (3 patients; VAS 60-79), and the most severe pain imaginable (1 patient; VAS 80-100). The remaining patient, who had initiated burosumab treatment prior to the EAP, was pain-free (VAS 0).

Bone densitometry was performed at the time of the first burosumab injection or prior to treatment initiation in 5 of the 9 patients. In 3 patients, the assessment was conducted on the same day or within the preceding month, while for the other 2 patients, it was completed 4 to 9 mo earlier. Among the treatment-naive patients, 2 exhibited osteopenia at the lumbar spine, with *T*-scores between −2.5 and −1, while a third treatment-naive patient, who had the earliest examination, showed osteoporosis (*T*-score ≤ −2.5). At the total hip, osteopenia was found in 2 burosumab-naive patients, and osteoporosis was present in another burosumab-naive patient. In contrast, among the pre-exposed patients, 2 had osteopenia at the lumbar spine, while 1 pre-exposed patient exhibited osteoporosis at the total hip. The fifth patient, who had already been treated with burosumab prior to inclusion in the program, had a normal total hip *T*-score (>−1).

### Treatment duration and dosage

Follow-up visits were not available for all patients, as the visit dates serve as indicative rather than mandatory within the legal data collection period and are subject to patient availability and scheduling.

In the 7 patients who had at least 1 follow-up visit, the duration of burosumab treatment ranged from 5.7 to 12.1 mo up to the last known injection. The durations of burosumab treatment prior to enrollment in the EAP were as follows: patient 7, 21 mo; patient 8, 20 mo; and patient 9, 14 mo.

Importantly, there were no instances of transient discontinuation of treatment among these patients, nor were there any cases of permanent discontinuation.

**Figure 2:**
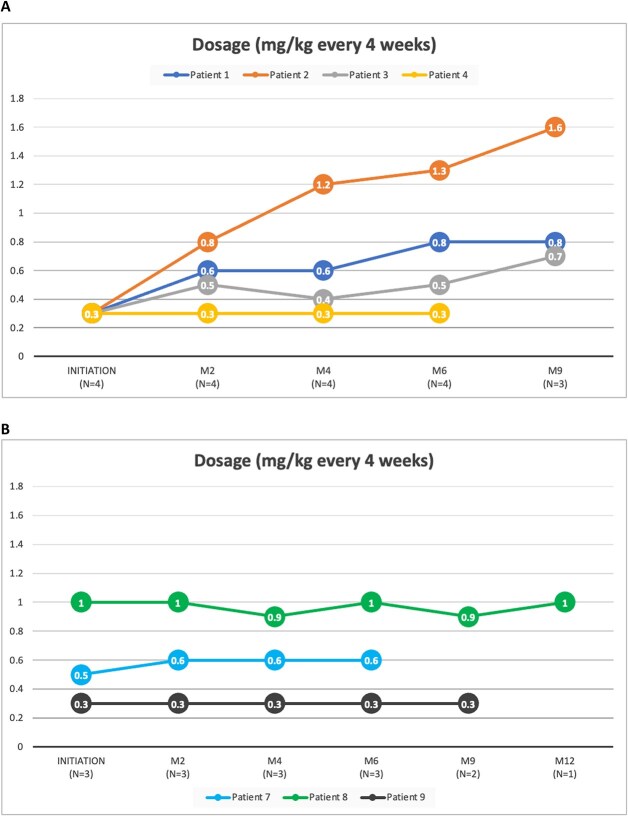
Burosumab treatment dosage during patient follow-up in (A) burosumab-naive patients and (B) pre-exposed patients. Patient 2 and patient 6 had an initial dosage value of 0.3, but no follow-up data regarding their dosage were available.

Figure 2 illustrates the burosumab treatment dosage during patient follow-up. For the naive patients (*n* = 6) depicted in Figure 2A, the treatment was initiated at 0.3 mg/kg every 4 wk, as per the protocol. Among these, 4 patients had at least 1 follow-up visit. In 3 of these patients, the dose per injection was increased due to persistently low phosphatemia levels. Figure 2B shows the burosumab treatment dosage for the pre-exposed patients (*n* = 3), with small fluctuations in dose per injection observed in 2 patients.

### Assessment of burosumab efficacy

The efficacy of burosumab treatment was evaluated based on changes in pain levels and phosphatemia.

#### Clinical evolution (pain)

**Figure 3:**
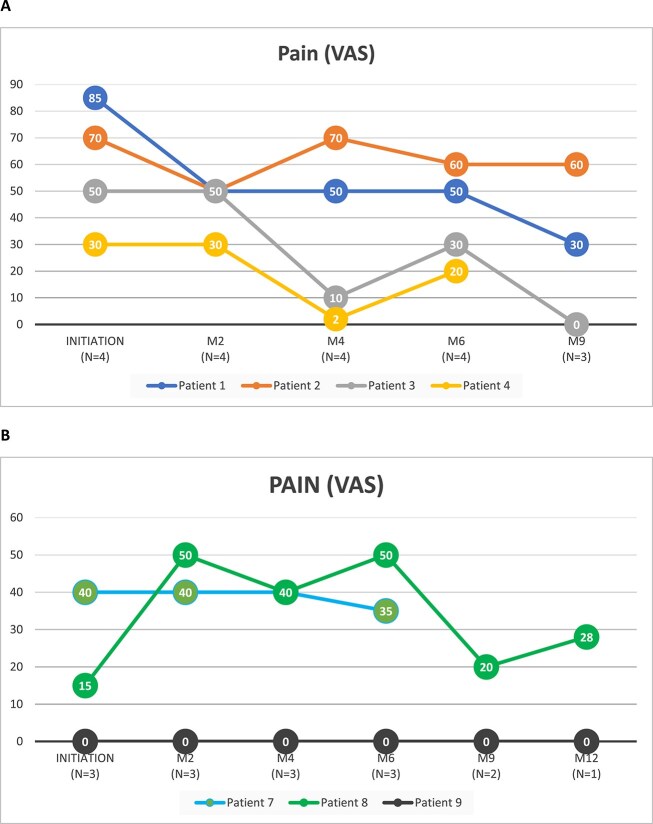
Pain evolution in (A) burosumab-naive and (B) pre-exposed patients. Abbreviation: VAS, visual analog scale. Patient 2 and patient 6 had an initial pain score of 30 and 40, respectively, but no follow-up data regarding their pain score was available.

Among the 4 burosumab-naïve patients with at least 1 follow-up visit after the initial injection, all reported a decrease in pain during each follow-up visit. For 2 of these patients (patients 1 and 3), the reduction in pain was substantial: 1 patient experienced a decrease from the most intense pain possible (VAS 80-100) to mild pain (VAS 1-39), and the other from moderate pain (VAS 40-59) to no pain (VAS 0; Figure 3A).

Among the 3 pre-exposed patients, pain levels remained stable in 2 patients (patients 7 and 9) and fluctuated in the third (patient 8; Figure 3B).

#### Serum phosphate assessment

**Figure 4:**
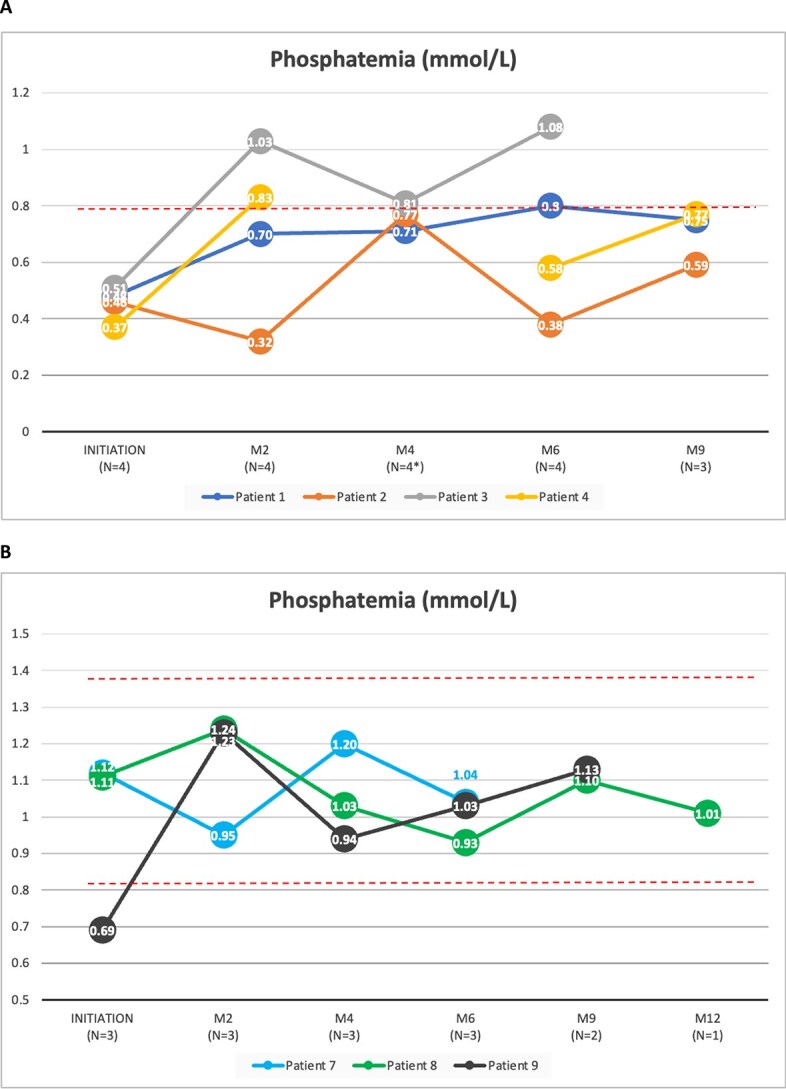
Serum phosphate levels in (A) burosumab-naive and (B) pre-exposed patients. The dotted lines indicate the phophatemia normal range (between 0.8 and 1.5 mmol/L.

In burosumab-naive patients, fasting serum phosphate levels were observed as follows: 1 patient (patient 3) achieved a level > 0.8 mmol/L, another patient (patient 1) showed an increase from 0.48 to 0.75 mmol/L at mo 9, while 2 patients (patients 2 and 4) experienced fluctuations in their phosphatemia levels, as depicted in Figure 4A. In pre-exposed patients, fasting serum phosphate fluctuated between 0.8 and 1.5 mmol/L until mo 9 or mo 12, as shown in Figure 4B.

### Safety

One non-serious, unexpected adverse event was reported in a pre-exposed patient. During a tumor re-evaluation, there was an incidental discovery of a significant increase in C-terminal FGF23 levels, rising from 210 RU/mL in August 2021 to 25 289 RU/mL in February 2022, and further to 81 000 RU/mL in February 2023 after inclusion in the program. Despite this increase, the patient continued burosumab treatment until the end of the follow-up.

### Quality of life

The RAPID-3 questionnaire was fully completed and thus analyzable by 7 out of the 9 patients at the start of treatment in the EAP. This group included 5 burosumab-naive patients and 2 patients already receiving burosumab. Additionally, 5 of these patients completed at least 1 follow-up questionnaire during the EAP, comprising 3 burosumab-naive patients and 2 pre-exposed patients. Another burosumab-naive patient, who did not have a RAPID-3 assessment at the initial burosumab injection, completed the questionnaire during subsequent follow-up visits.

**Figure 5:**
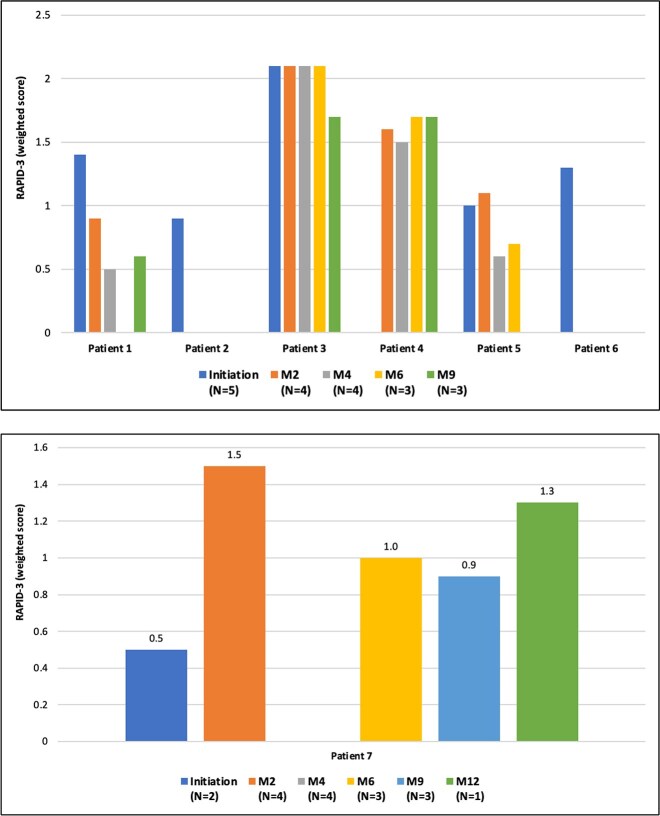
Patient quality of life, using the RAPID-3 questionnaire, in (A) burosumab-naive and (B) pre-exposed patients. The RAPID-3 weighted score ranges from 0 to 10; the lower the score, the better the quality of life. Patient 2 and patient 6 had a RAPID-3 score recorded at initiation but no data available throughout the follow-up period, while patient 7 had no data recorded at any point. Abbreviation: RAPID-3, Routine Assessment of Patient Index Data 3.

At the time of the initial burosumab injection, 3 of the 5 burosumab-naive patients had minimally affected QoL (weighted RAPID-3 score < 2.3), and 2 patients were in new remission (weighted score ≤ 1, indicating minimal symptom burden; Figure 5A). Both patients already on burosumab at inclusion were also in new remission at the start of the treatment (Figure 5B).

In burosumab-naive patients, the QoL remained largely unaffected throughout treatment initiation and follow-up assessments, with some patients showing either stability or new remission at each evaluation. In the pre-exposed group, RAPID-3 data was available for 2 patients during the follow-up period: 1 patient demonstrated minimal impact across all scores, while the other consistently reported no pain throughout the entire follow-up duration (Figure 5).

## Discussion

In this case series, the real-world efficacy and safety of burosumab in treating TIO was evaluated. Burosumab treatment in patients with TIO resulted in reduced pain and improved phosphatemia levels, with consistent treatment adherence and no serious adverse events reported. Quality of life assessments indicated minimal impairment in most patients at the start of treatment. Our findings provide valuable data to the limited existing literature on TIO management and highlight the potential benefits of burosumab for patients with this rare condition.

The primary clinical manifestations of TIO, including hypophosphatemia and musculoskeletal pain, were consistent with previous reports. Our results show that burosumab effectively alleviates pain in patients, as evidenced by the VAS scores. Notably, burosumab-naïve patients reported significant pain reduction, with some achieving minimal or no pain. This improvement aligns with findings from the UX023T-CL201 trial, suggesting that pain reduction is partly linked to potential fracture consolidation leading to decreased pain and fatigue.^9^

Serum phosphate levels improved in some patients following burosumab treatment, although not all achieved the targeted threshold of 0.8 mmol/L. This may be partially due to the initial burosumab dose being too low to effectively stabilize phosphatemia. The starting dose of 0.3 mg/kg is significantly lower than the initial dose of 1 mg/kg typically used for treating XLH. The normalization of serum phosphate in TIO patients is challenging, as evidenced by the variable responses in our cohort. The gradual increase in burosumab dosage, ranging from 0.2 to 0.6 mg/kg, depending on age and response to treatment, further complicates achieving rapid serum phosphate normalization. It often takes about a year for patients to reach the maximum authorized dose of 2 mg/kg in TIO patients, suggesting that higher initial doses or faster titration may be necessary for more immediate biochemical stabilization. In addition, while serum phosphate levels did not fully normalize in all patients, they showed clinical improvement consistent with findings from previously published Phase 2 trials. This aligns with the experience of clinicians using conventional treatments, such as phosphate and vitamin D analogs.

The variability in phosphatemia response among patients highlights the considerable heterogeneity in TIO presentations and patient characteristics. Differences in disease severity, tumor localization, and individual metabolic responses can all influence treatment outcomes. This patient heterogeneity underscores the need for personalized treatment approaches and careful monitoring to optimize therapeutic efficacy.

Additionally, future studies should evaluate serum phosphatemia levels at peak timepoints (1-2 wk post-administration) to capture the initial responsiveness to burosumab. Additionally, long-term monitoring of osteomalacia improvement should prioritize bone-specific alkaline phosphatase as a marker over serum phosphatemia levels.

Quality of life, assessed using the RAPID-3 questionnaire, showed improvement in some patients during the EAP. While the limited number of patients and follow-up assessments preclude definitive conclusions, the trends observed suggest that burosumab might have a positive impact on HR-QoL, comparable to the results observed in the phase 2 trials.

In addition, these results may be attributed to the relatively good baseline HR-QoL of the pre-exposed group, who had already been treated with burosumab and, therefore, experienced better quality of life compared to burosumab-naive patients. Their longer exposure to treatment and prior improvements could explain the minimal perceived impact or further improvement. Furthermore, habituation to the disease and developed resilience over time may have also contributed to this outcome across all patients.

These findings highlight the importance of regular monitoring and comprehensive assessment in managing TIO patients.

Notably, the consistent adherence to burosumab treatment, with no discontinuations among the 7 patients over 5.7 to 12.1 mo, highlights its favorable tolerability and manageable safety profile. This finding suggests that burosumab is a viable long-term treatment option in this patient cohort.

The safety profile of burosumab in our cohort was consistent with previous studies, with no new safety concerns identified, with the exception of a non-serious adverse event in an individual patient. In 1 pre-exposed patient, a significant increase in C-terminal FGF23 levels was observed during follow-up. It is important to note that FGF23 levels should not be measured while a patient is receiving burosumab, as the assay detects the burosumab molecule bound to FGF23, leading to interference in the measurement. This likely explains the observed rise in C-terminal FGF23 levels and indicates that the increase is not an unresolved adverse event, but rather a result of assay interference caused by the presence of burosumab.

The favorable safety and efficacy outcomes in this real-world setting support burosumab as a viable therapeutic option for TIO patients, particularly those with inoperable tumors or those unresponsive to conventional treatments.

We acknowledge that 1 limitation of our study is the relatively short follow-up period for a majority of the patients. Among the 9 exposed patients, 7 had attended at least 1 follow-up visit (7 patients at mo 2, 4, and 6; 5 at mo 9; and 1 at mo 12). Thus, we have a follow-up of a majority of the patients only until 6 mo. This limitation is linked to the EAP procedure, which ends on the same date for all patients, corresponding to the reimbursement date in the indication concerned. This provides a good initial evaluation of the benefits of burosumab but underscores the need for further and long-term investigations to fully understand its efficacy and safety profile.

Additionally, the results related to HR-QoL may reflect the choice of the RAPID-3 questionnaire, which might not fully capture the QoL in this specific patient population. This questionnaire was selected in accordance with the authorities’ guidelines, as required for the EAP. Despite these limitations, our findings offer meaningful clinical insights into this rare pathology, helping to address the lack of real-life data in this context.

## Conclusion

Our case series provides initial real-world evidence on the efficacy and safety of burosumab in treating TIO. Burosumab appears to alleviate pain and stabilize serum phosphate levels in the majority of patients in our cohort, suggesting potential improvements in QoL. These findings are consistent with previous clinical trial results, highlighting burosumab as a valuable therapeutic option for managing TIO. A key strength of our study lies in its standardized approach, which supports the reliability of our findings within a diverse patient group. Moreover, since some patients had initiated treatment prior to their inclusion in the program, our study provides insights that may better reflect the long-term efficacy and safety of burosumab. However, further studies with larger patient cohorts and extended follow-up periods are needed to confirm these early observations and to establish a more comprehensive understanding of the long-term safety and efficacy profile of burosumab.

## Data Availability

The datasets supporting the conclusions of this article are available from the corresponding author upon reasonable request.
